# A novel assay for screening inhibitors targeting HIV-1 integrase dimerization based on Ni-NTA magnetic agarose beads

**DOI:** 10.1038/srep25375

**Published:** 2016-05-03

**Authors:** Dawei Zhang, Hongqiu He, Mengmeng Liu, Zhixia Meng, Shunxing Guo

**Affiliations:** 1 Institute of Medicinal Plant Development, Chinese Academy of Medical Sciences & Peking Union Medical College, Beijing, 100193, China; 2 Institute of Bioinformatics and Medical Engineering, School of Electrical and Information Engineering, Jiangsu University of Technology, Changzhou, 213001, China; 3 Chongqing Center for Biomedicines and Medical Equipment, Chongqing Academy of Science and Technology, Chongqing, 401123, China.

## Abstract

Human immunodeficiency virus (HIV)-1 integrase (IN), which mediates integration of viral cDNA into the cellular chromosome, is a validated antiviral drug target. Three IN inhibitors, raltegravir, elvitegravir and dolutegravir, have been clinically approved since 2008. However, drug resistance have emerged in infected patients receiving treatment using these drugs which share the same mechanism of action and have a low genetic barrier for resistance. Therefore, there is an urgent need to develop drugs with novel mechanism. IN requires a precise and dynamic equilibrium between several oligomeric species for its activities. The modulation of the process which is termed as IN oligomerization, presents an interesting allosteric target for drug development. In this research, we developed a magnetic beads based approach to assay the IN dimerization. Then, using the assay we screened a library of 1000 Food and Drug Administration (FDA)-approved drugs for IN dimerization inhibitors and identified dexlansoprazole as a potential IN dimerization inhibitor. In conclusion, the assay presented here has been proven to be sensitive and specific for the detection of IN dimerization as well as for the identification of antiviral drugs targeting IN dimerization. Moreover, a FDA-approved proton-pump inhibitors, dexlansoprazole, was identified as a potential inhibitor for IN dimerization.

Retroviruses such as HIV-1 are characterized by integration of reverse-transcribed viral genome into the host cell chromosome^1^. Viral integration, which is catalyzed by HIV-1 integrase (IN), comprises two spatially and temporally distinct steps, 3′ processing and strand transfer^2^. As a critical enzyme in the viral life cycle, IN is currently targeted by three FDA-approved drugs: raltegravir (RAL), elvitegravir (EVG) and dolutegravir (DTG)^3^. All these drugs have the same mechanism of action: blocking the strand transfer activity of IN and are collectively termed as IN strand transfer inhibitors (INSTIs). However, significant cross-resistance has been observed within INSTIs in infected patients receiving treatment^4^,^5^,^6^,^7^. As a consequence, there is an urgent need to develop novel drugs with mechanism distinct from INSTIs to avoid existing and emerging multi-drug resistant HIV-1 strains.

IN is found as an equilibrium of monomers, dimers, tetramers, and even higher multimeric forms during integration, which is termed as IN oligomerization^8^. IN dimerization has been shown to be a plausible therapeutic target, for which several compounds and peptides have been found to display inhibitory activity^9^.

Recently, an AlphaScreen technology-based method for screening IN dimerization inhibitor was reported. However, this method has an obvious limitation: the requirement of expensive and sophisticated instruments which are not available to all laboratories. Moreover, a homogeneous time-resolved fluorescence based (HTRF) assay for detection of IN dimerization was reported and used to study the dynamics of IN dimerization^11^. However, to the best of our knowledge, this assay has not been validated for high-throughput screening (HTS) or used for the screening of inhibitors targeting IN.

Drug repositioning is the process of identifying new uses for drugs outside the scope of their original medical indication^12^. By exploiting existing knowledge of drugs, drug repositioning can offer a faster and cheaper approach than traditional drug discovery^13^. Drug repositioning has become an increasingly important part of the drug development landscape, with many pharmaceutical and biotech companies now having repositioning programs^14^. With lower costs, shorter development times and higher success rates, drug repositioning is also ideally suited for academia-based drug discovery^14^.

In this study, we developed a novel IN dimerization assay. Using the method, we undertook a drug repositioning screen to identify unknown IN dimerization inhibitory activity for known drugs. Besides, to provide confidence in our hits during screening, we implemented a counterscreen to eliminate molecules that interfere with the screening method itself.

## Results and Discussion

### Principle of the assay

**Figure 1:**
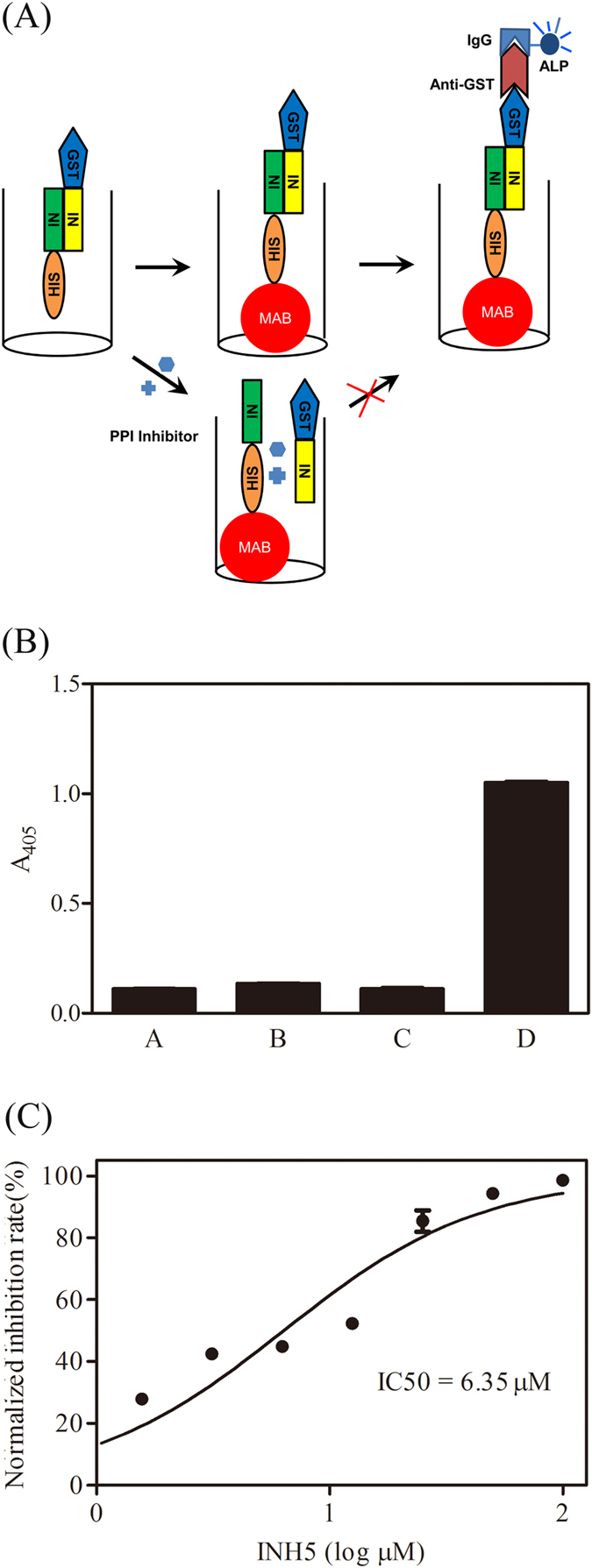
Development of an *in vitro* screening method to identify IN dimerization inhibitors. (**A**) The schemes depict the principle of the assay for IN dimerization. GST–tagged IN (yellow) is mixed with His_6_-tagged IN (green) at the desired concentrations. Incubation at room temperature allows the formation of GST-IN/His_6_-IN heterodimers as well as GST-IN and His_6_-IN homodimers. Then, heterodimers will be captured by Ni^2+^ -coated magnetic beads (red) through C-terminal His_6_-tag and detected by alkaline phosphatase conjugated anti-GST antibody (dark red) through its N-terminal GST-tag. Changes in the population of heterodimers due to the presence of modulating compounds will result in an altered output signal and can be picked up. (**B**) Assay performance under optimal reaction conditions. Bars (**A–C**) represent signals produced from negative controls in the absence of either GST-IN and His6-IN or GST-IN or His6-IN respectively. Bar D represent signals produced from IN dimerization. (**C**) Inhibition of IN dimerization by peptide INH5. Error bars represent SD from 3 replicate values.

The principle of the method is illustrated in Fig. 1A. In the assay, GST-tagged IN (yellow) is mixed with His_6_-tagged IN (green) at the desired concentrations. Incubation at room temperature allows the formation of GST-IN/His_6_-IN heterodimers as well as GST-IN and His_6_-IN homodimers. Then, heterodimers will be captured by Ni^2+^ -coated magnetic beads (red) through C-terminal His_6_-tag and detected by alkaline phosphatase conjugated anti-GST antibody (dark red) through its N-terminal GST-tag. Whereas, neither of two kind of homodimers will be captured by Ni^2+^ -coated magnetic beads and detected by alkaline phosphatase conjugated anti-GST antibody simultaneously. Hence, GST-IN/His_6_-IN heterodimers will be detected as IN dimerization by the assay. The presence of modulating compounds in the assay will change the population of heterodimers or block the formation of them, resulting in an altered output signal and can be picked up by the spectrophotometer.

### Assay development, performance evaluation and validation

This study aimed to establish a high-throughput assay for screening inhibitors targeting IN dimerization. To optimize the assay, titration experiments were performed. The optimal concentration of GST-IN and His_6_-IN was determined to be 20 nM respectively. The primary antibody was prepared at a 1/4000 and secondary antibody at 1/5000. IN has the propensity to aggregate^15^, and theoretically IN tetramers and higher order oligomers containing both GST- and His_6_-tags may also be captured by magnetic beads and detected by alkaline phosphatase conjugated anti-GST antibody in the assay. Generally, we cannot fully exclude that only IN dimers contribute to signal generation. However, in previous studies, the dissociation constants of IN dimers and tetramer were determined to be 67.8 pM and 22 μM^11^,^16^. In their research, Tsiang *et al*. suggested that at the integrase concentrations used a maximum of 67.5 nM, the presence of integrase tetramer is negligible (0.068% of total integrase)^11^. Accordingly, in our study, both GST-IN and His_6_-IN were used at 20 nM, and the total concentration of IN was 40 nM which was less than 67.5 nM used by Tsiang *et al*. Therefore, in our assay, most of the IN existed in the form of dimer, the presence of IN tetramer and multimer should be omitted for simplicity.

Z′ factor has been defined to evaluate suitability of an assay for HTS and signal-to-noise ratio (S/N), signal-to-background ratio (S/B) are widely used to indicate the quality of an assay^17^,^18^,^19^. To test the suitability and quality of the assay, we measured the signal of all reactions under optimal reaction conditions. As shown in Fig. 1B, the negative controls in the absence of either GST-IN and His6-IN or GST-IN or His6-IN showed background readings measured as absorbance at 405 nm (A_405_), lower than 0.11. Wheras, the IN dimerization gave a signal of 1.05. Accordingly, we determined the S/N, S/B and z′ factor values of the assay and found that S/N, S/B and z′ factor values was 295, 10 and 0.98 respective, which all exceeded the minimum criteria (S/N > 10, S/B > 3 and z′ > 0.5). Taken together, these values of statistical parameters confirmed that the assay developed was high sensitivity, specificity, and was suitable to be developed for HTS.

Peptide INH5 (ATDQAEHLKTAVQMAVFIHNYKA-CONH2), which was previously reported as an inhibitor of IN dimerization, was used as a probe to test whether the HTS assay was effective for drug screening targeting IN dimerization^20^. As shown in Fig. 1C, INH5 inhibited the IN dimerization with an IC_50_ of 6.35 μM (95% confidence interval, 2.45 μM to 16.47 μM) which was are comparable to previous experiment data 4.50 μM, indicating that the assay can robustly detect decreases in signal and is therefore effective and suitable for a HTS application^10^.

### Identifying IN dimerization inhibitory activities for existing drugs

**Figure 2:**
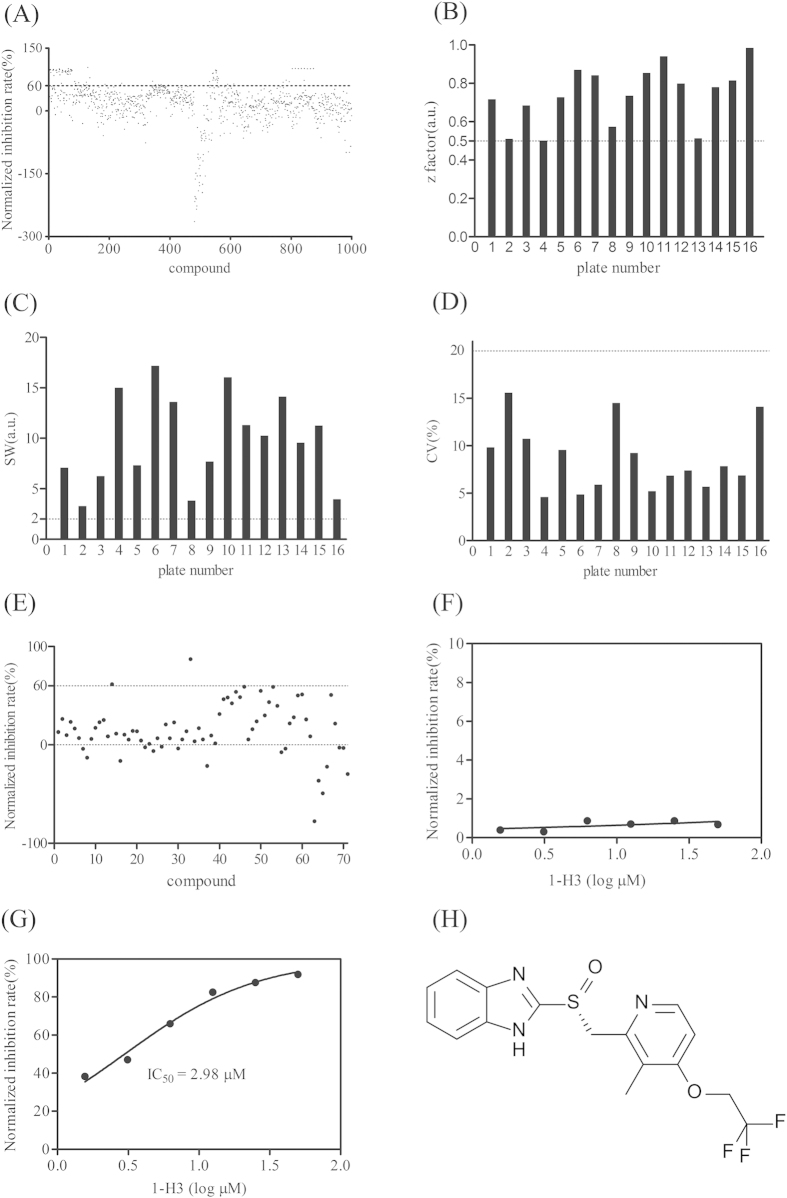
Results of the drug repositioning screen for IN dimerization inhibitors. (**A**) Scatterplots of the primary screening using the developed assay. Dashed line shows the ‘hits’ selection criteria (>60% disruption of a PPI complex); z′ factor (**B**), SW (**C**)and CV (**D**) of each plate in the primary screening. Dashed lines show the minimum pass criteria (z′ > 0.5, S/N > 10, S/B > 3, SW > 2, CV < 20%). (**E**) Scatterplots of the confirmatory screening using the developed assay. Dashed line shows the ‘hits’ selection criteria (>60% disruption of a PPI complex). Z′ factor, SW and CV of the confirmatory screening were 0.99, 6.19 and 10.8% respectively. (**F**) Counterscreen using His_6_-GST protein was used to analyze the interference between dexlansoprazole and the assay system; (**G**) Dexlansoprazole identified in the screening exhibited a sigmoidal dose-dependent reduction of assay signal; (**H**) The chemical structure of dexlansoprazole.

The optimized IN dimerization assay was used to screen a partial library of pharmacologically active compounds for inhibitors of IN dimerization. Compounds were screened as described in materials and methods section, with appropriate comparison controls included on each plate. Hits were scored as compounds that showed an inhibition ratio of the signal higher than 60% in the assays. The z′, signal window (SW), and coefficient of variation (CV) of each plate were calculated and then compared with the minimum pass criteria (z′ > 0.5, SW > 2.0, CV < 20%) to evaluate the quality of the HTS data. In a primary screening, at the concentration of 30 μM, 71 of the 1000 initial compounds were identified as hits, with a hit rate of 7.1% (Fig. 2A). All HTS campaigns in the primary screening exceeded the minimum criteria (z′ > 0.5, SW > 2, and CV < 20%) (Fig. 2B–D), indicating that the results of the screening were reliable and could be used for further investigated. Then, 71 compounds screened out from the primary screening were re-screened and only one compound, dexlansoprazole (Fig. 2H), showed an excellent inhibitory rate of 87.17% (Fig. 2E). All HTS campaigns in the confirmatory screening also exceeded the minimum criteria (z′ > 0.5, SW > 2, and CV < 20%), as such dexlansoprazole could be used for further study.

### Non-specificity counterscreen validation

In a previous study, Schorpp *et al*. suggested that in a drug screening system using His_6_-tag protein and nickel chelating beads, false-positive compounds could be identified^21^. This can be attributed to two possible reasons. One is hydrogen-bonded complexes formed between his-tagged protein and the compound would prevent immobilization of the protein on the beads surface. The other is the compound may act as nickel chelating agents and adsorb on the bead surface, thus preventing binding of the proteins to the beads surface. Thus, in this study, a nonspecific counterscreen was performed to confirm dexlansoprazole is a specific inhibitor for IN dimerization rather than a false positive interfering with the assay system. In the counterscreen assay, His_6_-GST protein was used, which could bind the Ni^2+^ coupling magnetic beads and be detected by GST antibody, generating an extremely strong signal. Any compound that interferes with beads or with the His_6_-tag of His_6_-GST protein will result in a decreased signal in the counterscreen assay. The results (Fig. 2F) showed that dexlansoprazole almost had no effect on the assay system, whereas displayed a dose-dependent effect on IN dimerization with an IC_50_ value of 2.98 μM (Fig. 2G). All these results indicate that dexlansoprazole is a specific small molecular inhibitor for IN dimerization.

## Conclusion

Based on Ni-NTA magnetic agarose beads, we have developed a highly sensitive and effective screening method to identify inhibitors of IN dimerization, and have successfully identified a FDA-approved drug dexlansoprazole as a potential small molecular inhibitor of IN dimerization, which may therefore serve as a lead compound for further optimization.

## Methods

### Construction of protein expression plasmids

The full-length HIV-1 IN coding sequence was amplified by PCR from pET28a-IN with a His_6_ fusion tag on its N-terminus (conserved in our lab). Then, the amplified IN gene was subcloned into *Nco* I-*Xho* I sites of the vector pET28a to create the pET28a-IN for expression of recombinant C-terminal His_6_–tagged IN protein (IN-His_6_). In a similar manner, the amplified IN gene was subcloned into the *Bam*H I-*Sal* I sites of the vector pGEX-4 T-1, creating pGEX-4T-IN for expression of recombinant N-terminal GST-tagged IN protein (GST-IN). The pGEX-4T-His_6_ plasmid for expression of recombinant C-terminal His_6_-tagged GST (His_6_-GST) used for counterscreen was constructed by inserting the His_6_-tag encoding sequences (CACCACCACCACCACCACTGA) to the *Bam*H I-*Sal* I sites of the plasmid pGEX-4T-1.

### Recombinant proteins expression and purification

IN-His_6_ was expressed and purified by affinity chromatography as previously described^22^. GST-IN and double-tagged GST-His_6_ protein were produced using standard conditions as previously described^10^. The concentration of the all protein was determined by the Bradford assay with bovine serum albumin (BSA) as a standard. 12% SDS-PAGE was performed to determine the purity of the proteins.

### The IN dimerization assay

The IN dimerization assay was developed based on Ni-NTA magnetic agarose beads. The assay was performed in 96-well NBS microplate in a final volume of 100 μl per well. Briefly, the assay works as follows. Compounds and proteins were diluted to 10× and 5× working solutions in the 1× assay buffer (25 mM tris-HCl, pH 7.4, 150 mM NaCl, 1 mM MgCl_2_, 0.05% BSA[v/w]) respectively. First, 10 μl compound dilution, 50 μl 1× buffer, 20 μl each of protein dilutions were pipetted into the wells. The plate was sealed and left to incubate at 200 rpm for 3 h at room time (RT). Next, the 10 μl magnetic beads were added followed by incubating at 900 rpm for 30 min at RT. Then, the plate was placed on a magnetic concentrator, the supernatant was discarded, and the wells were washed 3 times with 200 μl wash buffer (50 mM sodium phosphate, pH 7.4, 150 mM NaCl, 0.005% Tween-20[v/v], 20 mM imidazole). The wells were incubated with 100 μl mouse anti-GST antibody diluted 1:4000 in antibody dilution buffer (50 mM sodium phosphate, pH 7.4, 150 mM NaCl, 0.005% Tween-20[v/v], 0.5%[w/v]BSA) at 900 rpm for 30 min at 37 °C, followed by washing three times as above. The wells were incubated with 100 μl rabbit anti-mouse IgG alkaline phosphatase conjugated antibody diluted 1:5000 in antibody dilution buffer at 900 rpm for 30 min at 37 °C, followed by washing three times as above. The color reaction was developed with 100 μl substrate buffer (10 mM diethanolamine, pH 9.8, 0.5 mM MgCl_2_, 8 mM p-NPP) for 30 min at 37 °C. The plate was read at 405 nm with an Enspire multimode reader (PerkinElmer, USA). The reproducibility, signal stability, and robustness were determined for PPI assay to ensure it was HTS compatible. Also one IN dimerization inhibitor INH5 was used to validate the assay for HTS^10^.

### Library screening for inhibitors of IN dimerization

In total, 1000 compounds were selected at random from ‘The Spectrum Collection’ purchased from National compound resource center. The library, which is composed of 2,320 compounds in 10 mM DMSO solution, lists primarily FDA-approved human therapeutic drugs, drug-like compounds, and natural products. Each compound was diluted in DMSO to reach a concentration of 300 μM, which was used as the 10× compound stock. In all screening campaigns, no-protein negative controls (including GST-IN was omitted or IN-His was omitted or both protein were omitted) and the positive control contained 10% v/v DMSO only. The negative and positive control with 8 replicates wells each located randomly among the 96 wells of each plate. The high-throughput assay was carried out in replicate in 96-well microplates as above.

A counterscreen was carried out as described above, replacing purified IN-His_6_ and GST-IN with 20 nM His_6_-tagged GST protein.

### Data Analysis

The z′ factor, signal-to-noise ratio (S/N), signal-to-background ratio (S/B), signal window (SW), and coefficient of variation (CV) were calculated and then compared with the minimum pass criteria (z′ > 0.5, S/B > 3, SW > 2, CV < 20%)^21^. The ‘hits’ for PPI assays were classified as compounds that led to > 60% disruption of a PPI complex. All HTS data were processed using Excel (Microsoft Corp.) and visualized using Prism 5.0 (GraphPad Software).

## Additional Information

**How to cite this article**: Dawei, Z. *et al.* A novel assay for screening inhibitors targeting HIV-1 integrase dimerization based on Ni-NTA magnetic agarose beads. *Sci. Rep.* **6**, 25375; doi: 10.1038/srep25375 (2016).

## References

[b1] Craigie R. & Bushman F. D. H. I. V. DNA integration. Cold Spring Harb Perspect Med. 2, a006890 (2012). 22762018 10.1101/cshperspect.a006890 PMC3385939

[b2] Craigie R. The molecular biology of HIV integrase. Future Virol. 7, 679–89 (2012). 23024700 10.2217/FVL.12.56 PMC3458710

[b3] Hazuda D. J. HIV integrase as a target for antiretroviral therapy. Curr Opin HIV AIDS 7, 383–89 (2012). 22871634 10.1097/COH.0b013e3283567309

[b4] Malet I. *et al.* New raltegravir resistance pathways induce broad cross-resistance to all currently used integrase inhibitors. J Antimicrob Chemoth. 69, 2118–2112 (2014). 10.1093/jac/dku095 24710029

[b5] Sichtig N. *et al.* Evolution of raltegravir resistance during therapy. J Antimicrob Chemother. 64, 25–32 (2009). 19447792 10.1093/jac/dkp153

[b6] Steigbigel R. T. *et al.* Raltegravir with optimized background therapy for resistant HIV-1 infection. N Engl J Med. 359, 339–54 (2008). 18650512 10.1056/NEJMoa0708975

[b7] Métifiot M. *et al.* Elvitegravir overcomes resistance to raltegravir induced by integrase mutation Y143. AIDS 25, 1175–78 (2011). 21505303 10.1097/QAD.0b013e3283473599 PMC7380719

[b8] Hayouka Z. *et al.* Inhibiting HIV-1 integrase by shifting its oligomerization equilibrium. Proc Natl Acad Sci USA 104, 8316–21 (2007). 17488811 10.1073/pnas.0700781104 PMC1895947

[b9] Feng L., Larue R. C., Slaughter A., Kessl J. J. & Kvaratskhelia M. Curr Top Microbiol Immunol 389, 1–27 (2015). 25778682 10.1007/82_2015_439 PMC4791179

[b10] Demeulemeester J., Tintori C., Botta M., Debyser Z. & Christ F. Development of an AlphaScreen-based HIV-1 integrase dimerization assay for discovery of novel allosteric inhibitors. J Biomol Screen. 17, 618–28 (2012). 22337657 10.1177/1087057111436343

[b11] Tsiang M. *et al.* Affinities between the binding partners of the HIV-1 integrase dimer-lens epithelium-derived growth factor (IN dimer-LEDGF) complex. J Bio Chem. 284, 33580–99 (2009). 19801648 10.1074/jbc.M109.040121 PMC2785201

[b12] Krantz A. Protein-site targeting. Diversification of the drug discovery process. Nat Biotechnol. 16, 1294 (1998). 9853592 10.1038/4243

[b13] Jin G. & Wong S. T. C. Toward better drug repositioning: prioritizing and integrating existing methods into efficient pipelines. Drug Discov Today 19, 637–44 (2014). 24239728 10.1016/j.drudis.2013.11.005 PMC4021005

[b14] Ashburn T. T. & Thor K. B. Drug repositioning: identifying and developing new uses for existing drugs. Nat Rev Drug Discov. 3, 673–83 (2004). 15286734 10.1038/nrd1468

[b15] Guiot E. *et al.* Relationship between the Oligomeric Status of HIV-1 Integrase on DNA and Enzymatic Activity. J Biol Chem. 281, 22707–19 (2006). 16774912 10.1074/jbc.M602198200

[b16] Jenkins T. M., Engelman A., Ghirlando R. & Craigie R. A. Soluble active mutant of HIV-1 integrase: involvement of both the core and carboxyl-terminal domains in multimerization. J Biol Chem. 271, 7712–18 (1996). 8631811 10.1074/jbc.271.13.7712

[b17] Inglese J. *et al.* High-throughput screening assays for the identification of chemical probes. Nat Chem Biol. 3, 466–79 (2007). 17637779 10.1038/nchembio.2007.17

[b18] Birmingham A. *et al.* Statistical methods for analysis of high-throughput RNA interference screens. Nat Methods 6, 569–75 (2009). 19644458 10.1038/nmeth.1351 PMC2789971

[b19] Zhang J. H., Chung T. D. Y. & Oldenburg K. R. A simple statistical parameter for use in evaluation and validation of high throughput screening assays. J Biomol Screen., 4, 67–73 (1999). 10838414 10.1177/108705719900400206

[b20] Maroun R. G. *et al.* Peptide inhibitors of HIV-1 integrase dissociate the enzyme oligomers. Biochemistry 40, 13840–48 (2001). 11705373 10.1021/bi011328n

[b21] Schorpp K. *et al.* Identification of small-molecule frequent hitters from AlphaScreen high-throughput screens. J Biomol Screen. 19, 715–26 (2014). 24371213 10.1177/1087057113516861 PMC4153540

[b22] He H. Q. *et al.* A novel high-throughput format assay for HIV-1 integrase strand transfer reaction using magnetic beads. Acta Pharmacol Sin. 29, 397–404 (2008). 18298906 10.1111/j.1745-7254.2008.00748.x

